# Supplementary material for: Iron phthalocyanine on Au(111) is a “non-Landau” Fermi liquid
Source: Nat Commun. 2021 Oct 15;12:6027. doi: 10.1038/s41467-021-26339-z (PMC8521586; doi:10.1038/s41467-021-26339-z)
Supplement: Supplementary file 1 — Supplementary info [file 41467_2021_26339_MOESM1_ESM.pdf]

Supplementary Information - Iron phthalocyanine on Au(111) is a "non-Landau" Fermi liquid

R. Žitko
Jožef Stefan Institute, Jamova 39, SI-1000 Ljubljana, Slovenia and
Faculty of Mathematics and Physics, University of Ljubljana, Jadranska 19, SI-1000 Ljubljana, Slovenia

G. G. Blesio
Instituto de Física Rosario (CONICET) and Universidad Nacional de Rosario,
Bv. 27 de Febrero 210 bis, 2000 Rosario, Argentina and
Jožef Stefan Institute, Jamova 39, SI-1000 Ljubljana, Slovenia

L. O. Manuel
Instituto de Física Rosario (CONICET) and Universidad Nacional de Rosario,
Bv. 27 de Febrero 210 bis, 2000 Rosario, Argentina

A. A. Aligia
Centro Atómico Bariloche and Instituto Balseiro,
Comisión Nacional de Energía Atómica and CONICET, 8400 Bariloche, Argentina

Supplementary Note 1: Auxiliary multiorbital two-channel Anderson model

In order to analyze the topological features of the $S = 1$ Kondo model (1) with two non-equivalent channels, encoded in the generalized Friedel sum rules that we will deduce in Supplementary Note 2, it is better to work with the auxiliary multiorbital two-channel Anderson Hamiltonian,

$$H_A = H_0 + H_{\mathrm{int}}. \tag{S1}$$

H_0 contains the one-body terms:

$$H_0 = H_{\mathrm{cond}} + H_{\mathrm{hyb}} + H_d. \tag{S2}$$

H_{cond} corresponds to two conduction bands (channels): one with symmetry $3z^2 - r^2$ ($\tau = 1$) and another that takes into account the degenerate π-manifold xz, yz ($\tau = -1$)[1]:

$$H_{\mathrm{cond}} = \sum_{k\tau\sigma} \varepsilon_{k\tau} c^\dagger_{k\tau\sigma} c_{k\tau\sigma}; \tag{S3}$$

we consider conduction electron energy dispersions such that both bands have the same constant density of states (DOS) and we work in the wide-band limit, such that the half-bandwidth W is the largest energy scale in the problem.

H_{hyb} contains the hybridization between the conduction and impurity states with the same τ symmetry:

$$H_{\mathrm{hyb}} = \sum_{k\tau} \left(V_\tau c^\dagger_{k\tau\sigma} d_{\tau\sigma} + \mathrm{H.c.} \right). \tag{S4}$$

For correct description of FePc on Au(111), it is essential to allow for very different coupling strenghts V_τ of each localized orbital with the substrate, giving rise to a multiorbital Anderson model with non-equivalent orbitals. Due to its spatial dependence, the $\tau = 1$ localized orbital hybridizes stronger with the gold surface than the $\tau = -1$ orbital. As a consequence, $V_1 > V_{-1}$.

H_d includes the impurity single-orbital (hole) energies and the Zeeman term:

$$H_d = \sum_{\tau\sigma} \epsilon_\tau n_{\tau\sigma} - BS_z \equiv \sum_{\tau\sigma} \epsilon_{\tau\sigma} n_{\tau\sigma}, \;\; \text{with} \;\; \epsilon_{\tau\sigma} \equiv \epsilon_\tau - \sigma \frac{B}{2}. \tag{S5}$$

We consider a Zeeman term only at the impurity site because for constant conduction-band DOS in the wide-band limit, the magnetic field has no effect on the conduction band (i.e., it can be eliminated through a simple energy shift).

The interaction terms act on the impurity states:

$$H_{\rm int} = \sum_\tau U_\tau n_{\tau\uparrow}n_{\tau\downarrow} - J_H \boldsymbol{S}_1 \cdot \boldsymbol{S}_{-1} + D\left(\sum_\tau S_{z\tau}\right)^2. \tag{S6}$$

We study this model in the particle-hole symmetric case, corresponding to $\epsilon_\tau = -\frac{U_\tau}{2}$ and symmetric conduction bands relative to the $\omega = 0$ Fermi level) in order to have exactly one hole in each impurity orbital. In the Kondo regime, $-\epsilon_\tau \gg V_\tau^2/W$, these two holes are ferromagnetically coupled by the first Hund's rule, $J_H > 0$, yielding an effective $S = 1$ at the impurity site. Due to symmetry considerations of the involved 3d orbitals, we take the same Hubbard repulsion U for each impurity orbital. This implies that both single-orbital energies ϵ_τ should be degenerate in the particle-hole symmetric case, while *ab initio* calculations[2] give an energy difference of the order of 1 eV. In the Kondo regime this difference is irrelevant.

Among the interactions of a generic multiorbital Anderson Hamiltonian for 3d impurities[3], we are neglecting the inter-orbital Hubbard repulsion $U' n_1 n_{-1}$, as this term is almost constant in the (particle-hole symmetric) Kondo regime. We also neglect the pair hopping interaction $J_H(d^\dagger_{1\uparrow}d^\dagger_{1\downarrow}d_{-1\downarrow}d_{-1\uparrow} + \text{H.c.})$, as, for FePc on Au(111), the two-electron low energy sector contains only the triplet states due to the strong Hund's coupling[2]. While the inter-orbital Hubbard repulsion U' could be easily included in the NRG computation, the pair hopping interaction breaks the separate conservation of the isospin in each channel τ, resulting in a very demanding computational effort.

By means of the equation of motion method, it can be shown[4] that all the conduction band information needed for the computation of impurity observables is encoded in the hybridization functions

$$\Delta_\tau(\omega) = \pi \sum_k V_\tau^2 \delta(\omega - \varepsilon_{k\tau}). \tag{S7}$$

As we assume equal constant conduction-band DOS for boths channels and energy-independent V_τ, the hybridisation functions Δ_τ are likewise constant functions. This is a very reliable assumption for normal conduction bands with a finite DOS at the Fermi level, as in this case the Kondo physics depend only marginally on the exact form of the DOS. It should be stressed that, if we were interested in substrate observables like STM spectra far from the impurity, there would be a need for a more thorough modeling of the conduction bands and the tunneling parameters V_τ.

It is worth to mention that, in the Kondo regime, the Anderson Hamiltonian (S1) can be exactly mapped to the Kondo Hamiltonian (1) by means of the Schrieffer-Wolff transformation[4], with Kondo exchange interactions given by $J_\tau = 4V_\tau^2/U$. Potential scattering is absent due to the particle-hole symmetry. We have numerically verified the agreement between the predictions for both Hamiltonians. Although the Kondo model is simpler because of its reduced local Hilbert space, it is better to work with the auxiliar Hamiltonian (S1) in order to clearly interpret the Friedel sum rules, which involves the computation of the localized spin-orbital occupancies $\langle n_{\tau\sigma}\rangle$.

Conservation laws for the Anderson Hamiltonian

The Hamiltonian H_A commutes with the total electron number *per* orbital

$$N_\tau = \sum_{k\sigma} n_{k\tau\sigma} + \sum_\sigma n_{\tau\sigma} \tag{S8}$$

($n_{k\tau\sigma} \equiv c^\dagger_{k\tau\sigma}c_{k\tau\sigma}$) and with the total electron number *per* spin

$$N_\sigma = \sum_{k\tau} n_{k\tau\sigma} + \sum_\tau n_{\tau\sigma}. \tag{S9}$$

Consequently, the system conserves the total electron number,

$$N = \sum_{\tau\sigma} N_{\tau\sigma}, \tag{S10}$$

the total spin projection along the z direction,

$$S_z^{\rm tot} = \frac{1}{2}\sum_{\tau\sigma} \sigma N_{\tau\sigma}, \tag{S11}$$

and the total orbital isospin

$$T_z = \sum_{\tau\sigma} \tau N_{\tau\sigma}. \tag{S12}$$

As there are no spin-orbit couplings and the magnetic field B is along the single-ion anisotropy axis z, the Hamiltonian H_A also commutes with the total spin

$$\boldsymbol{S}^2 = \frac{1}{2}\left(S_+S_- + S_-S_+\right) + \left(S_z\right)^2, \tag{S13}$$

where $S_+ = \sum_\tau \left(\sum_k c^\dagger_{k\tau\uparrow} c_{k\tau\downarrow} + d^\dagger_{\tau\uparrow} d_{\tau\downarrow}\right)$, $S_- = (S_+)^\dagger$. It is worth to mention that if the single-particle energies $\varepsilon_{k\tau\sigma}$ and $\epsilon_{\tau\sigma}$ in H_0 were arbitrary, the total spin $\boldsymbol{S}^2$ would commute with H_A only if the relations $\varepsilon_{\mu\tau\uparrow} - \varepsilon_{\mu\tau\downarrow} = \varepsilon_{\mu'\tau'\uparrow} - \varepsilon_{\mu'\tau'\downarrow}$ hold for all $\mu, \mu' = k, d; \tau, \tau' = 1, -1$.

Supplementary Note 2: Generalized Friedel sum rules

Green's functions and electron occupation numbers

Under the conservation laws (S11), (S12), it can be shown by means of the Lehmann representation that all the single-particle Green's functions (GF) of H_A ,

$$G^{kk'}_{\tau\sigma\tau'\sigma'} \equiv \ll c_{k\tau\sigma} | c^\dagger_{k'\tau'\sigma'} \gg, \quad G^{kd}_{\tau\sigma\tau'\sigma'} \equiv \ll c_{k\tau\sigma} | d^\dagger_{\tau'\sigma'} \gg,$$

$$G^{dk'}_{\tau\sigma\tau'\sigma'} \equiv \ll d_{\tau\sigma} | c^\dagger_{k'\tau'\sigma'} \gg, \quad G^{d}_{\tau\sigma\tau'\sigma'} \equiv \ll d_{\tau\sigma} | d^\dagger_{\tau'\sigma'} \gg,$$

are diagonal in spin and orbital indices. Thus, the GF matrix is block diagonal:

$$\mathbb{G} = \begin{pmatrix} \mathbb{G}_{1\uparrow} & 0 & 0 & 0 \\ 0 & \mathbb{G}_{1\downarrow} & 0 & 0 \\ 0 & 0 & \mathbb{G}_{-1\uparrow} & 0 \\ 0 & 0 & 0 & \mathbb{G}_{-1\downarrow} \end{pmatrix}, \tag{S14}$$

where the block indices run over the conduction electron momenta and the impurity, that is, over the set $I \equiv \{k_1, k_2, \cdots, d\}$.

As the interaction terms involve only the impurity degrees of freedom, we can express the conduction-band and mixed Green's functions in terms of the impurity GF G^d using the equations of motion. We have

$$\begin{aligned} G^{kk'}_{\tau\sigma} &= g_{k\tau} + |V_\tau|^2 g_{k\tau} G^d_{\tau\sigma} g_{k'\tau}, \\ G^{kd}_{\tau\sigma} &= V_\tau g_{k\tau} G^d_{\tau\sigma}, \\ G^{dk'}_{\tau\sigma} &= V^*_\tau g_{k'\tau} G^d_{\tau\sigma}, \end{aligned} \tag{S15}$$

where

$$g_{k\tau} = \frac{1}{z - \varepsilon_{k\tau}}. \tag{S16}$$

The total electron numbers *per* spin and *per* orbital can be expressed as the complex-plane contour integrals[5]

$$\langle N_{\tau\sigma} \rangle = \oint_\Gamma \frac{dz}{2\pi i} n_F(z) \mathrm{Tr}\ \mathbb{G}_{\tau\sigma}(z), \tag{S17}$$

where n_F is the Fermi function and the contour Γ, shown in Supplementary Fig. 1, encloses all the poles of the GF matrix (along the real axis) and none of the poles of n_F (along the imaginary axis). Using (S14) and (S15), we get

$$\mathrm{Tr}\ \mathbb{G}_{\tau\sigma}(z) = \sum_k G^{kk}_{\tau\sigma}(z) + G^d_{\tau\sigma}(z) = \sum_k g_{k\tau} + \sum_k \frac{|V_\tau|^2}{(z - \varepsilon_{k\tau})^2} G^d_{\tau\sigma}(z) + G^d_{\tau\sigma}(z). \tag{S18}$$

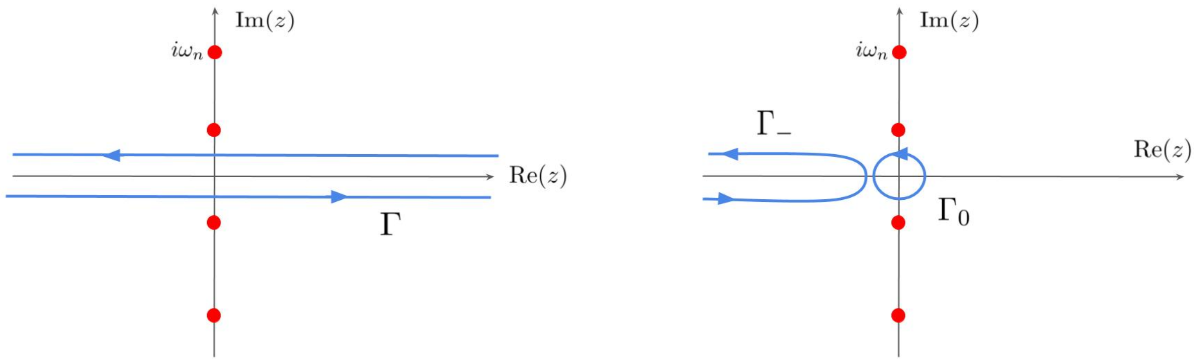

Supplementary Fig. 1: **Integration contours in the complex plane. a)** Γ encloses all the real axis; **b)** Γ_0, the origin of the complex plane, and Γ_-, the negative real axis. The dots represent the fermionic Matsubara frequencies $i\omega_n = i\frac{(2n+1)\pi}{\beta}$.

If we define the complex hybridization function

$$\Gamma_\tau(z) \equiv \sum_k \frac{|V_\tau|^2}{z - \varepsilon_{k\tau}}, \tag{S19}$$

then

$$\mathrm{Tr}\, \mathbb{G}_{\tau\sigma}(z) = \sum_k \frac{1}{z - \varepsilon_{k\tau}} + \left(1 - \frac{\partial \Gamma_\tau(z)}{\partial z}\right) G^d_{\tau\sigma}(z). \tag{S20}$$

Now we re-express the trace of the GF matrix in terms only of impurity functions (GF and self-energy). Using the formula[16]

$$\det \mathbb{G}_{\tau\sigma} = \left(\prod_k g_{k\tau}\right) G^d_{\tau\sigma}, \tag{S21}$$

and the impurity Dyson equation

$$(G^d_{\tau\sigma})^{-1} = z - \epsilon_{\tau\sigma} - \Gamma_\tau(z) - \Sigma^d_{\tau\sigma}(z), \tag{S22}$$

where $\Sigma^d_{\tau\sigma}$ is the impurity self-energy due to the interaction terms (S6), we obtain

$$\frac{\partial \ln \det \mathbb{G}^{-1}_{\tau\sigma}}{\partial z} = \sum_k \frac{\partial \ln g^{-1}_{k\tau}}{\partial z} + \frac{\partial \ln (G^d_{\tau\sigma})^{-1}}{\partial z} = \sum_k g_{k\tau} + \left(1 - \frac{\partial \Gamma_\tau}{\partial z} - \frac{\partial \Sigma^d_{\tau\sigma}}{\partial z}\right) G^d_{\tau\sigma}. \tag{S23}$$

Therefore,

$$\mathrm{Tr}\, \mathbb{G}_{\tau\sigma} = \frac{\partial \ln \det \mathbb{G}^{-1}_{\tau\sigma}}{\partial z} + G^d_{\tau\sigma}(z) \frac{\partial \Sigma^d_{\tau\sigma}(z)}{\partial z}, \tag{S24}$$

and the electron numbers are given by

$$\langle N_{\tau\sigma} \rangle = \oint_\Gamma \frac{dz}{2\pi i} n_F(z) \frac{\partial \ln \det \mathbb{G}^{-1}_{\tau\sigma}}{\partial z} + \oint_\Gamma \frac{dz}{2\pi i} n_F(z) G^d_{\tau\sigma}(z) \frac{\partial \Sigma^d_{\tau\sigma}(z)}{\partial z}. \tag{S25}$$

Generalized Friedel sum rules and Luttinger integrals

Here, we generalize the Friedel sum rules for multiorbital models using the conservation laws studied by Yoshimori and Zawadowski[6]. The first integral in the Supplementary Eq. (S25) for the electron numbers can be rewritten, using (S23), as

$$\sum_k \oint_\Gamma \frac{dz}{2\pi i} n_F(z) g_{k\tau}(z) + \oint_\Gamma \frac{dz}{2\pi i} n_F(z) \frac{\partial \ln (G^d_{\tau\sigma})^{-1}}{\partial z} \equiv \langle N^{c(0)}_{\tau\sigma} \rangle + \oint_\Gamma \frac{dz}{2\pi i} n_F(z) \frac{\partial \ln (G^d_{\tau\sigma})^{-1}}{\partial z}, \tag{S26}$$

where $\langle N^{c(0)}_{\tau\sigma}\rangle$ are the conduction electron numbers in the absence of the impurity. In the wide-band limit, the number of conduction electrons remains unaltered when the impurity is introduced. As a consequence $\langle N_{\tau\sigma}\rangle - \langle N^{c(0)}_{\tau\sigma}\rangle = \langle n_{\tau\sigma}\rangle$. On the other hand, the last integral in (S26), in the zero temperature limit, is

$$\lim_{T\to 0}\oint_\Gamma \frac{dz}{2\pi i} n_F(z) \frac{\partial \ln (G^d_{\tau\sigma})^{-1}}{\partial z} = -\frac{1}{\pi}\mathrm{Im}\int_{-\infty}^{0}\frac{\partial \ln (G^d_{\tau\sigma})^{-1}}{\partial \omega}d\omega = \frac{1}{\pi}\mathrm{Im}\ln G^d_{\tau\sigma}(0) - \frac{1}{\pi}\mathrm{Im}\ln G^d_{\tau\sigma}(-\infty) = \frac{\delta_{\tau\sigma}}{\pi}, \quad \text{(S27)}$$

where we have defined the (Fermi level) phase shift $\delta_{\tau\sigma}$[7] through

$$G^d_{\tau\sigma}(0) = -|G^d_{\tau\sigma}(0)|e^{i\delta_{\tau\sigma}}.$$

Putting all these ingredients together we obtain the generalized Friedel sum rules, that relate the quasiparticle phase shifts and the impurity occupancies at zero T,

$$\delta_{\tau\sigma} = \pi\langle n_{\tau\sigma}\rangle + I_{\tau\sigma}, \quad \text{(S28)}$$

with the Luttinger integrals $I_{\tau\sigma}$[8] defined as

$$I_{\tau\sigma} = \mathrm{Im}\int_{-\infty}^{0} G^d_{\tau\sigma}\frac{\partial \Sigma^d_{\tau\sigma}}{\partial\omega}d\omega. \quad \text{(S29)}$$

Until very recently, it had been considered that the vanishing of the Luttinger integrals represents one of the hallmarks of Fermi liquid phases[7,8]. In this way, the (ordinary) Friedel sum rules state that the quasiparticle phase shifts are simply the impurity occupancies divided by π, as it was known from the fifties. However, in the last two years, it was shown that, for certain single-impurity[9,10] and two-impurity[11,12] models, the Luttinger integrals can take the discrete values $\pm\pi/2$ in the so-called *non-Landau* Fermi liquids (NLFL), that is, Fermi liquid phases that are not adiabatically connected with their non-interacting counterparts[9]. In this work we have found that an iron phthalocyanine molecule on Au(111) behaves as a NLFL in the absence of an applied magnetic field, while for $B \neq 0$ it is an "ordinary" Fermi liquid, but with non-zero Luttinger integrals that change continuosly with B. So, we have shown that there are Fermi liquids in which the Friedel sum rules (S28) holds only in its generalized version with non-zero (continuous) Luttinger integrals.

As the impurity spectral function at the Fermi level is

$$\rho_{\tau\sigma}(0) = -\frac{1}{\pi}\mathrm{Im}G^d_{\tau\sigma}(0) = \frac{1}{\pi}|G^d_{\tau\sigma}(0)|\sin(\delta_{\tau\sigma}), \quad \text{(S30)}$$

for a Fermi liquid whose low (but *finite*) energy behavior is characterized by $\mathrm{Im}\Sigma^d_{\tau\sigma} \propto -a\omega^2$ $(a > 0)$. This results in $|G^d_{\tau\sigma}(\omega \to 0)| = \sin\delta_{\tau\sigma}/\Delta_\tau$. Consequently, the Friedel sum rule for spectral functions, valid for Fermi liquids, is given by

$$\rho_{\tau\sigma}(0) = \frac{1}{\pi\Delta_\tau}\sin^2\delta_{\tau\sigma}. \quad \text{(S31)}$$

The above argument remains valid if the imaginary part of the self-energy has a Dirac δ centered exactly at the Fermi level, as it happens in the NLFL[9] where $\mathrm{Im}\Sigma^d_{\tau\sigma} = -a\omega^2 - b\delta(\omega)$. The generalized Friedel sum rule for the spectral funcion $\rho_{\tau\sigma}(\omega \to 0)$ (S31) therefore holds even for the NLFL.

Topological interpretation of the Luttinger integrals

Here, we demonstrate that for certain Anderson impurity models (single-orbital, degenerate two-orbital with an arbitrary magnetic field, non-equivalent two-orbital model without magnetic field), the Luttinger integrals have a topological nature. For this purpose, we turn off the interactions of the Anderson Hamiltonian H_A. The total electron numbers can change as the interactions are turned off. Using (S25) the electron numbers in the non-interacting case are

$$\langle N_{\tau\sigma}\rangle^0 = \oint_\Gamma \frac{dz}{2\pi i} n_F(z)\frac{\partial \ln\det(\mathbb{G}^{(0)}{}_{\tau\sigma})^{-1}}{\partial z}, \quad \text{(S32)}$$

where $\mathbb{G}^{(0)}{}_{\tau\sigma}$ are the non-interacting GF block matrices. One should not confound these electron numbers, corresponding to the non-interacting Anderson model ($H = H_0$, i.e. setting $H_{\mathrm{int}} = 0$) with the impurity still hybridized

with the conduction channels, with the previously defined $\langle N^{c(0)}_{\tau\sigma}\rangle$ (Supplementary Eq. S26) that correspond to the conduction electron numbers for the case of decoupled impurity (i.e., setting $H_{\rm hyb}=0$).

From (S25) and (S32), we obtain

$$\langle N_{\tau\sigma}\rangle - \langle N_{\tau\sigma}\rangle^0 = \oint_\Gamma \frac{dz}{2\pi i} n_F(z)\frac{\partial \ln D_{\tau\sigma}}{\partial z} + \oint_\Gamma \frac{dz}{2\pi i} n_F(z) G^d_{\tau\sigma}(z)\frac{\partial \Sigma^d_{\tau\sigma}(z)}{\partial z}, \tag{S33}$$

where we have defined the scalar function

$$D_{\tau\sigma}(z) = \frac{\det \mathbb{G}^{(0)}{}_{\tau\sigma}}{\det \mathbb{G}_{\tau\sigma}}. \tag{S34}$$

Taking into account (S21), its expression is simplied to

$$D_{\tau\sigma}(z) = \frac{G^{d(0)}_{\tau\sigma}(z)}{G^d_{\tau\sigma}(z)}. \tag{S35}$$

For $T\to 0$, it can be shown[13] that the integral

$$n_D(\Gamma) \equiv \oint_\Gamma \frac{dz}{2\pi i} n_F(z)\frac{\partial \ln D_{\tau\sigma}}{\partial z} \tag{S36}$$

is a winding number. When $T\to 0$, the vanishing of the Fermi function for Re $z>0$ turns Γ into the curve $\Gamma_-\cup\Gamma_0$ that encircle only the negative real axis and the origin (see Supplementary Fig. 1), and

$$\begin{aligned} n_D(\Gamma) &\to n_D(\Gamma_-) + \frac{1}{2}n_D(\Gamma_0) = \\ &= \oint_{\Gamma_-}\frac{dz}{2\pi i}\frac{\partial \ln D_{\tau\sigma}(z)}{\partial z} + \frac{1}{2}\oint_{\Gamma_0}\frac{dz}{2\pi i}\frac{\partial \ln D_{\tau\sigma}(z)}{\partial z} = \\ &= \frac{1}{2\pi i}\oint_{\mathcal{D}_{\Gamma_-}}\frac{dD_{\tau\sigma}}{D_{\tau\sigma}} + \frac{1}{2}\times\frac{1}{2\pi i}\oint_{\mathcal{D}_{\Gamma_0}}\frac{dD_{\tau\sigma}}{D_{\tau\sigma}}, \end{aligned} \tag{S37}$$

where $\mathcal{D}_{\Gamma_-}$ ($\mathcal{D}_{\Gamma_0}$) is the closed curve that describes $D(z)$, as z goes along Γ_- (Γ_0) in the complex plane (Re D, Im D). $n_D(\Gamma_-), n_D(\Gamma_0)$ are the winding numbers of D around the origin for paths Γ_- and Γ_0, respectively. These numbers are integer (positive if D winds counterclockwise around the origin, negative in the other direction). The 1/2 factor that multiply $n_D(\Gamma_0)$ comes from $n_F(z=0)=\frac{1}{2}$.

In the $T\to 0$ limit we can use the relations[5]

$$\lim_{T\to 0}\oint_\Gamma \frac{dz}{2\pi i} n_F(z)F(z) = -\frac{1}{\pi}\mathrm{Im}\int_{-\infty}^0 F(\omega)d\omega \tag{S38}$$

to get, from (S33),

$$\langle N_{\tau\sigma}\rangle - \langle N_{\tau\sigma}\rangle^0 = -\frac{1}{\pi}\mathrm{Im}\int_{-\infty}^0 \frac{\partial \ln D_{\tau\sigma}}{\partial \omega}d\omega - \frac{1}{\pi}I_{\tau\sigma}. \tag{S39}$$

The integral can be expressed in terms of winding numbers (S37), that is,

$$\langle N_{\tau\sigma}\rangle - \langle N_{\tau\sigma}\rangle^0 = n_D(\Gamma_-) + \frac{1}{2}n_D(\Gamma_0) - \frac{1}{\pi}I_{\tau\sigma}. \tag{S40}$$

From this we deduce that if the total electron numbers do not change when the interactions are turned off, the Luttinger integrals only take discrete values that are multiples of $\pi/2$:

$$I_{\tau\sigma} = \pi n_D(\Gamma_-) + \frac{\pi}{2}n_D(\Gamma_0). \tag{S41}$$

Note that $n_D(\Gamma_0)$ is different from zero if $D(z)$ has a zero or a pole at $z=0$. For example, if $D(z)=z$ or $1/z$ and we take Γ_0 as a circle of radius R centered in the origin, then

$$n_D(\Gamma_0) = \frac{1}{2\pi i}\lim_{R\to 0}\oint_{\Gamma_0(R)}\frac{dD}{D} = \frac{1}{2\pi i}\int_0^{2\pi}\frac{(\pm i)Re^{\pm i\theta}d\theta}{Re^{\pm i\theta}} = \pm 1. \tag{S42}$$

Effective one-body Hamiltonian

If, as it can in general be expected, $\langle N_{\tau\sigma}\rangle \neq \langle N_{\tau\sigma}\rangle^0$, we can try to tune the effective impurity one-body energies (chemical potential, magnetic field, crystal-field) in order to force the same interacting and non-interacting electron numbers. If this is possible, we will show that the Luttinger integrals keep their topological nature. We define an effective one-body impurity Hamiltonian $\tilde{H}_d$

$$\tilde{H}_d = \sum_{\tau\sigma} \left(\tilde{\epsilon} - \frac{\sigma}{2}\tilde{B} + \tau\tilde{C}\right) n_{\tau\sigma} \equiv \sum_{\tau\sigma} \tilde{\epsilon}_{\tau\sigma} n_{\tau\sigma}. \tag{S43}$$

The dependence of the single-particle energy $\tilde{\epsilon}_{\tau\sigma}$ on σ and τ is dictated by the requirement that the effective Hamiltonian obey the same conservation laws (see Supplementary Note 1) as the original Anderson Hamiltonian.

To go further we re-arrange the non-interacting and interacting terms of the impurity Hamiltonian:

$$H_{\text{imp}} = H_d + H_{\text{int}} = \tilde{H}_d + \tilde{H}_{\text{int}}, \tag{S44}$$

with

$$\tilde{H}_d = \sum_{\tau\sigma} \tilde{\epsilon}_{\tau\sigma} n_{\tau\sigma} = H_d + \sum_{\tau\sigma} \left(\tilde{\epsilon}_{\tau\sigma} - \epsilon_{\tau\sigma}\right) n_{\tau\sigma}, \tag{S45}$$

while

$$\tilde{H}_{\text{int}} = H_{\text{int}} + \sum_{\tau\sigma} \left(\epsilon_{\tau\sigma} - \tilde{\epsilon}_{d\tau\sigma}\right) n_{\tau\sigma}. \tag{S46}$$

As the Hamiltonian remains unaltered, the impurity Green function does not change. However, there is a rearrangement of its "non-interacting" and "interacting" contributions:

$$(G^d_{\tau\sigma})^{-1} = (G^{d(0)}_{\tau\sigma})^{-1} - \Sigma^d_{\tau\sigma} = z - \epsilon_{\tau\sigma} - \Gamma_\tau - \Sigma^d_{\tau\sigma} = \tag{S47}$$

$$= z - \tilde{\epsilon}_{\tau\sigma} - \Gamma_\tau - \Sigma^d_{\tau\sigma} + (\tilde{\epsilon}_{\tau\sigma} - \epsilon_{\tau\sigma}) = (\tilde{G}^{d(0)}_{\tau\sigma})^{-1} - \tilde{\Sigma}^d_{\tau\sigma}, \tag{S48}$$

where the renormalized self-energy is defined as the original one shifted by a constant real number (a Hartree contribution to the self-energy):

$$\tilde{\Sigma}^d_{\tau\sigma} = \Sigma^d_{\tau\sigma} + (\tilde{\epsilon}_{\tau\sigma} - \epsilon_{\tau\sigma}). \tag{S49}$$

We can see that the renormalization does not change the Luttinger integrals

$$\tilde{I}_{\tau\sigma} = \text{Im} \int_{-\infty}^{0} G^d_{\tau\sigma} \frac{\partial \tilde{\Sigma}^d_{\tau\sigma}}{\partial\omega} d\omega = I_{\tau\sigma}, \tag{S50}$$

as the constant shift of the self-energy is irrelevant due to the derivative.

Therefore, if we are able to tune the effective parameters so that all the "effective non-interacting" electron numbers coincide with the interacting ones, that is

$$\langle N_{\tau\sigma}\rangle = \langle \tilde{N}_{\tau\sigma}\rangle^0, \tag{S51}$$

then, the topological relation between the Luttinger integrals and the winding numbers

$$I_{\tau\sigma} = -\text{Im} \int_{-\infty}^{0} \frac{\partial \ln \tilde{D}_{\tau\sigma}}{\partial\omega} d\omega = \pi n_{\tilde{D}}(\Gamma_-) + \frac{\pi}{2} n_{\tilde{D}}(\Gamma_0), \tag{S52}$$

is still valid, with

$$\tilde{D}_{\tau\sigma} = \frac{\tilde{G}^{d(0)}_{\tau\sigma}}{G^d_{\tau\sigma}}. \tag{S53}$$

The possibility to satisfy the electron number conditions $\langle N_{\tau\sigma}\rangle = \langle \tilde{N}_{\tau\sigma}\rangle^0$ for an Anderson Hamiltonian by tuning the effective non-interacting parameters, depends in how many conditions we must enforce, and how many effective

parameters we have at our disposal for this purpose. For a single orbital Anderson model under a magnetic field, there are only two electron conduction numbers conditions, i.e. Summplementary Eq. (S51) for $\sigma=\uparrow$ and $\sigma=\downarrow$, to satisfy and we can use two effective parameters $(\tilde{\epsilon},\tilde{B})$. Therefore, the conditions can be satisfied and the Luttinger integrals have a topological nature. For a degenerate two-orbital Anderson model, even under a magnetic field, $\langle N_{1\sigma}\rangle=\langle N_{-1\sigma}\rangle$ always holds due to the degeneracy. As a consequence, there are again two electron conduction numbers conditions, and both can be satisfied by tuning the effective parameters. Finally, for the Anderson model corresponding to FePc on Au(111), there are two non-equivalent orbitals. If $B=0$, the spin symmetry $\langle N_{\tau\uparrow}\rangle=\langle N_{\tau\downarrow}\rangle$ reduces the electron number conditions to two, and, hence, they can be satisfied. However, in the presence of a finite magnetic field, there are four different electron number conditions $\langle N_{\tau\sigma}\rangle=\langle\tilde{N}_{\tau\sigma}\rangle^0$ with only three parameters at our disposal $(\tilde{\epsilon},\tilde{B},\tilde{C})$. Therefore, it would be impossible to make this fit for all the electron numbers. In this last case, the individual Luttinger integrals may not have a topological (discrete) nature. In fact, numerically we have found that $I_{\tau\sigma}$ change continuosly with B, and that this behavior is essential to understand the experimental STM spectra of FePc on Au(111) under a magnetic field.

Even though each individual Luttinger integral loses its topological nature for the non-equivalent two-channel Anderson Hamiltonian (S1), in the next section, we will demonstrate that, due to the conservation laws, certain linear combinations of Luttinger integrals do have a topological character.

Conservation laws and topological numbers

As already mentioned, for the non-degenerate two-orbital Anderson model, it is impossible to match the interacting and non-interacting electron number for each spin-orbital separately. However, with the help of the **three** effective parameters: chemical potencial $\tilde{\epsilon}$, magnetic field $\tilde{B}$, and crystal field $\tilde{C}$, using the non-interacting GF's corresponding to the effective non-interacting Anderson Hamiltonian

$$\tilde{G}^{d(0)}_{\tau\sigma}=\frac{1}{\omega+i0^{+}-\tilde{\epsilon}-\sigma\frac{\tilde{B}}{2}+\tau\tilde{C}-\Gamma_\tau(\omega)},$$

we can match, between the interacting and non-interacting models, the **three** conserved quantities (see Supplementary Note 1):

i) the total electron number N

$$N-\sum_{\tau\sigma}\langle N^{c(0)}_{\tau\sigma}\rangle\equiv\sum_{\tau\sigma}\langle n_{\tau\sigma}\rangle=\sum_{\tau\sigma}\langle\tilde{n}_{\tau\sigma}\rangle^0=-\frac{1}{\pi}\sum_{\tau\sigma}\arctan\left(\frac{\Delta_\tau}{\tilde{\epsilon}-\sigma\frac{\tilde{B}}{2}+\tau\tilde{C}}\right). \tag{S54}$$

ii) the total $S_z^{\rm tot}$

$$S_z^{\rm tot}=\frac{1}{2}\sum_{\tau\sigma}\sigma\langle n_{\tau\sigma}\rangle=\frac{1}{2}\sum_{\tau\sigma}\sigma\langle\tilde{n}_{\tau\sigma}\rangle^0=-\frac{1}{2\pi}\sum_{\tau\sigma}\sigma\arctan\left(\frac{\Delta_\tau}{\tilde{\epsilon}-\sigma\frac{\tilde{B}}{2}+\tau\tilde{C}}\right), \tag{S55}$$

and, iii) the total orbital isospin T_z

$$T_z\equiv N_1-N_{-1}\equiv\sum_{\tau\sigma}\tau\langle n_{\tau\sigma}\rangle=\sum_{\tau\sigma}\tau\langle\tilde{n}_{\tau\sigma}\rangle^0=-\frac{1}{\pi}\sum_{\tau\sigma}\tau\arctan\left(\frac{\Delta_\tau}{\tilde{\epsilon}-\sigma\frac{\tilde{B}}{2}+\tau\tilde{C}}\right). \tag{S56}$$

In this way, we can show that the following linear combination of Luttinger integrals

$$T\equiv\sum_{\tau\sigma}I_{\tau\sigma}=-\sum_{\tau\sigma}{\rm Im}\int_{-\infty}^{0}\frac{\partial\ln\tilde{D}_{\tau\sigma}}{\partial\omega}d\omega, \tag{S57}$$

$$T_\sigma\equiv\sum_{\tau\sigma}\sigma I_{\tau\sigma}=-\sum_{\tau\sigma}\sigma\,{\rm Im}\int_{-\infty}^{0}\frac{\partial\ln\tilde{D}_{\tau\sigma}}{\partial\omega}d\omega, \tag{S58}$$

$$T_\tau\equiv\sum_{\tau\sigma}\tau I_{\tau\sigma}=-\sum_{\tau\sigma}\tau\,{\rm Im}\int_{-\infty}^{0}\frac{\partial\ln\tilde{D}_{\tau\sigma}}{\partial\omega}d\omega, \tag{S59}$$

(S60)

are topological numbers, as the left hand integrals can be expressed in terms of winding numbers. By the numerical NRG computation of the Luttinger integrals, we have found that, for the model describing FePc on Au(111), $T_\sigma = T_\tau = 0$. Meanwhile T takes the non-trivial topological value 2π for $D > D_c$ and $B = 0$ and goes to zero when the magnetic field is turned on, signalling a topological transition from a NLFL to an OFL (with non-zero Luttinger integral) as a function of the applied magnetic field.

Supplementary Note 3: Magnetic field dependence of the impurity self-energy

Taking into account that $T_\tau = T_\sigma = 0$, the Luttinger integrals can be expressed as

$$I_{1\uparrow} = I_{-1\downarrow} = I_0 - \alpha(D, B), \quad I_{1\downarrow} = I_{-1\uparrow} = I_0 + \alpha(D, B), \tag{S61}$$

where $I_0 = T/4$ has a topological (discrete) nature, while $\alpha(D, B)$ is the non-topological component of the Luttinger integrals. For $B = 0$ ($D > D_c$) all the Luttinger integrals are equal, $I_{\tau\sigma} = \pi/2$. When a small positive B is turned on, $I_{-1\uparrow}(= I_{1\downarrow})$ changes continuosly from $\pi/2$ while $I_{1\uparrow}(= I_{-1\downarrow})$ has a jump to $-\pi/2$. For $B < 0$ these behaviors are reversed. The abrupt change in the Luttinger integrals can be ascribed to the shift of the $B = 0$ Fermi level pole of the imaginary part of the self-energies: for $\Sigma^d_{1\uparrow}, \Sigma^d_{-1\downarrow}$ a small positive B shifts (and broadens) the pole to negative energies of the order of $-B/2$, giving rise to a π change in the Luttinger integral. On the other hand, for $\Sigma^d_{1\downarrow}, \Sigma^d_{-1\uparrow}$, due to the symmetry $\mathrm{Im}\Sigma^d_{\tau\sigma}(\omega) = \mathrm{Im}\Sigma^d_{\tau-\sigma}(-\omega)$, the poles go to positive energies. These behaviors for $\mathrm{Im}\Sigma^d_{1\uparrow}, \mathrm{Im}\Sigma^d_{-1\downarrow}$ are displayed in Supplementary Fig. 2.

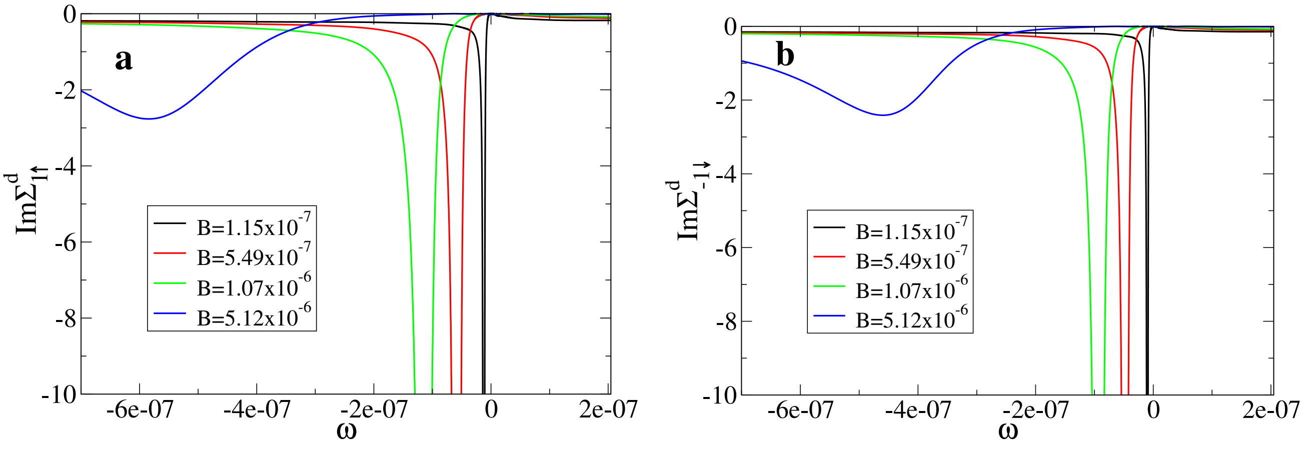

Supplementary Fig. 2: **Imaginary part of the self-energies.** **a)** $\Sigma^d_{1\uparrow}$ and **b)** $\Sigma^d_{-1\downarrow}$, close to the Fermi level, for different magnetic fields. The Anderson Hamiltonian parameters are the same as in the main text, except for $D = 1.08D_c$.

Supplementary Note 4: Detailed comparison with experiment

In this note, we compare side by side experimental results and our theory for three sets of experiments.

Dependence of the differential conductance as the molecule is separated from the surface

In Supplementary Fig. 3 we represent a set of experimental curves for the differential conductance dI/dV taken from Ref.[1] as the molecule is raised from the surface and compare them with the corresponding spectral density of the localized states of symmetry $3z^2 - r^2$ in our model. Since the experimental results are affected by a factor that decays exponentially with the distance between the tip and the surface, which is not included in our model, we have multiplied them by an arbitrary factor f.

In general, the shapes of experimental and theoretical curves are very similar, except for large separations (top) for which the theoretical curves are affected by the resolution of the NRG calculations and for small separations (bottom) for which some admixture of conduction states is expected to influence the conductance and is taken into account by

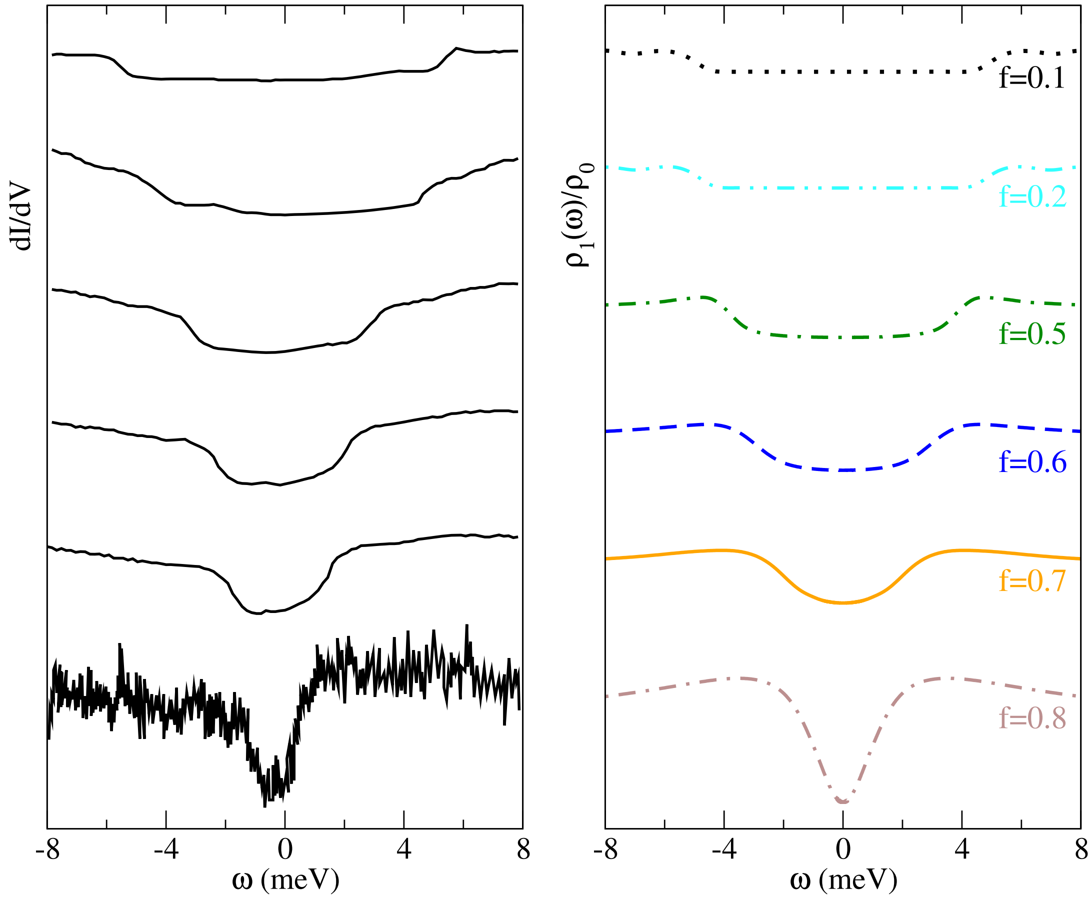

Supplementary Fig. 3: **Raising molecule from surface.** Left: experimental differential conductance as a function of voltage (taken from Fig. 2 (b) of Ref.[1]). Right: theoretical spectral density of level 1 as a function of energy, as the molecule is raised from the surface. The spectral densities plotted are the same as in Fig. 2 of the main text, where a rescaling along the vertical axis was made for a direct comparison with experimental data.

the factor q in Eq. (8) of the main text. In particular, the comparison when the molecule is on the surface is discussed below including this factor.

Differential conductance for different temperatures

In Supplementary Fig. 4 we compare experiment (taken mainly from Ref.[15]) and theory for the differential conductance dI/dV at different temperatures. The trend of experimental and theoretical curves is very similar. The theoretical curves have a steeper downwards slope for positive voltage, and in the experiment the maximum in dI/dV for positive voltage is larger than the relative maximum for negative voltage. Both features might be related with the effect of subtraction of a background or other features of a particular experiment. At the lowest experimental temperature (0.4 K) we also show the experimental curve taken from Fig. 1 (c) of Ref.[2]. This allows the reader to grasp some experimental uncertainties.

Differential conductance for different magnetic fields

In Supplementary Fig. 5 we compare the experimental results for dI/dV (also taken from Ref.[15]) and theory for different magnetic fields, assuming a gyromagnetic factor $g = 2$, inside the range of uncertainty of values reported in the supplemental material of Refs.[1],[15]. Taking into account the features mentioned above related with experimental

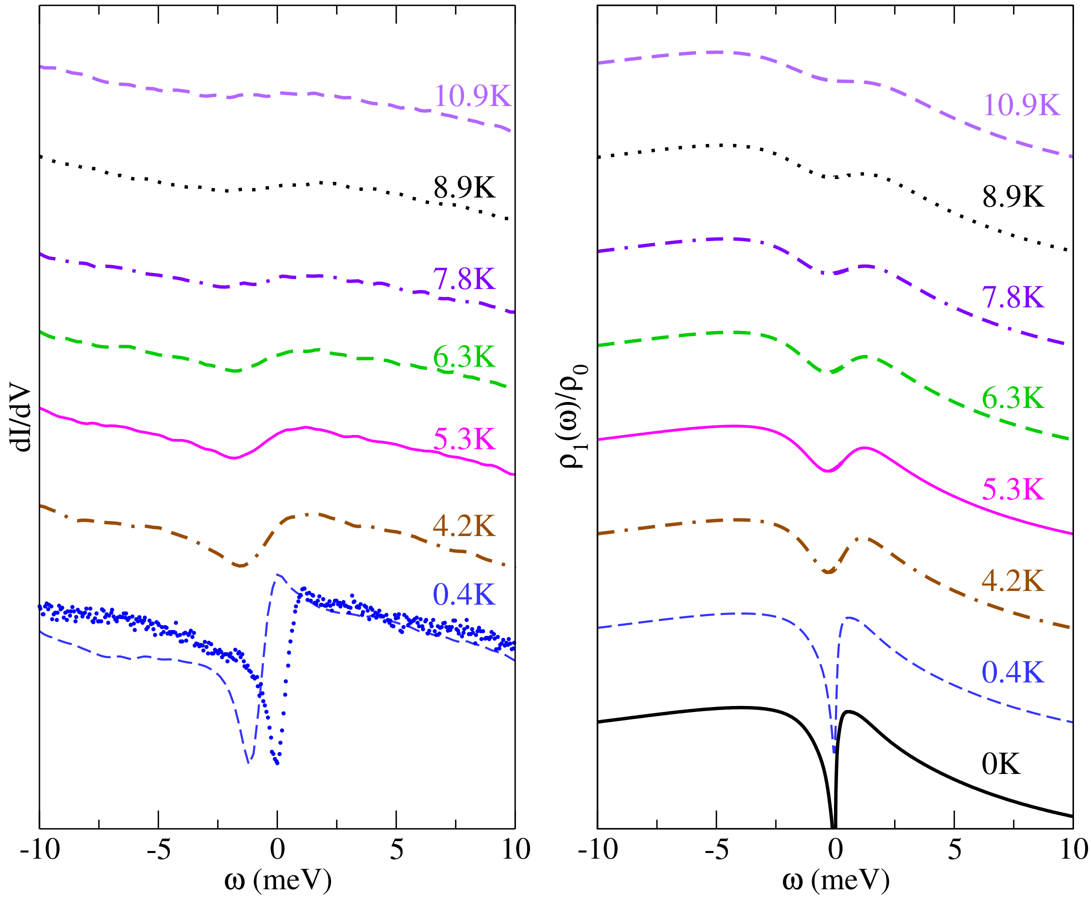

Supplementary Fig. 4: **Temperature dependence.** Left: experimental differential conductance as a function of voltage for several temperatures. Curves with different type of lines are taken from Fig. 2 (b) of Ref.[15] and data in dots ($T = 0.4\,K$) is taken with permission from Fig. 1 (c) of Ref.[2]. Right: temperature dependence of the differential conductance as a function of voltage as in Fig. 3 of the main text with a vertical displacement for a better comparison with the left panel.

uncertainties, and the absence of alternative physical explanations, the agreement is very good.

Supplementary References

[1] R. Hiraoka, E. Minamitani, R. Arafune, N. Tsukahara, S. Watanabe, M. Kawai, and N. Takagi, Single-molecule quantum dot as a Kondo simulator, Nature Commun. **8**, 16012 (2017).

[2] E. Minamitani, N. Tsukahara, D. Matsunaka, Y. Kim, N. Takagi, and M. Kawai, Symmetry-Driven Novel Kondo Effect in a Molecule, Phys. Rev. Lett. **109**, 086602 (2012).

[3] A. M. Oleš, Antiferromagnetism and correlation of electrons in transition metals, Phys. Rev. B **28**, 327 (1983).

[4] A. C. Hewson, *The Kondo Problem to Heavy Fermions*(Cambridge University Press, Cambridge, UK, 1997).

[5] G. Mahan, *Many-Particle Physics, Physics of Solids and Liquids* (Plenum, New York, 1990).

[6] A. Yoshimori and A. Zawadowski, Restricted Friedel sum rules and Korringa relations as consequences of conservation laws, Phys. C **15**, 5241 (1982).

[7] D. C. Langreth, Friedel Sum Rule for Anderson's Model of Localized Impurity States, Phys. Rev. **150**, 516 (1966).

[8] J. M. Luttinger, Fermi Surface and Some Simple Equilibrium Properties of a System of Interacting Fermions, Phys. Rev. **119**, 1153 (1960).

[9] G. G. Blesio, L. O. Manuel, P. Roura-Bas, and A. A. Aligia, Topological quantum phase transition between Fermi liquid phases in an Anderson impurity model, Phys. Rev. B **98**, 195435 (2018).

[10] G. G. Blesio, L. O. Manuel, P. Roura-Bas, and A. A. Aligia, Fully compensated Kondo effect for a two-channel spin $S = 1$ impurity, Phys. Rev. B **100**, 075434 (2019).

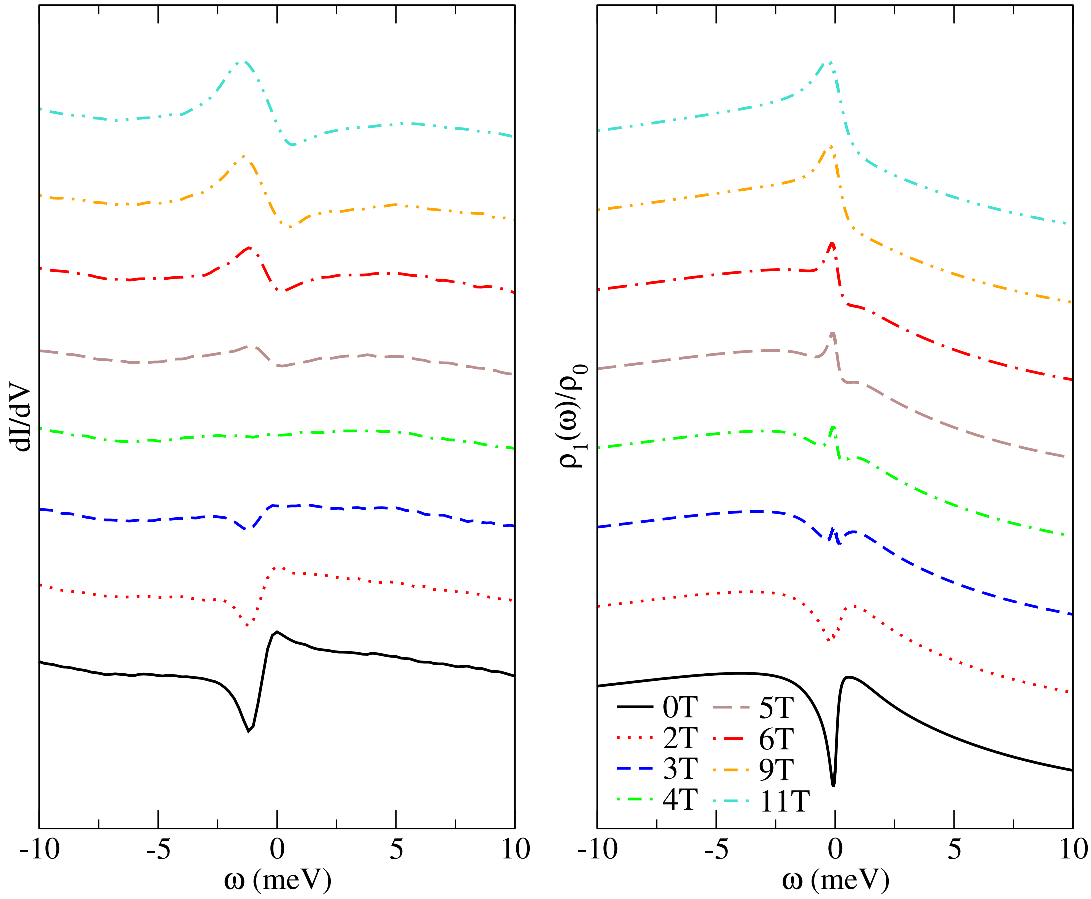

Supplementary Fig. 5: **Magnetic field dependence.** Left: experimental differential conductance as a function of voltage for several values of the magnetic field (taken from Fig. 2 (c) of Ref.[15]). Right: magnetic field dependence of the differential conductance as a function of voltage as in Fig. 4 of the main text with a vertical displacement for a better comparison with the left panel.

[11] O. J. Curtin, Y. Nishikawa, A. C. Hewson, and D. J. G. Crow, Fermi liquids and the Luttinger theorem, J. Phys. Commun. **2**, 031001 (2018).

[12] Y. Nishikawa, O. J. Curtin, A. C. Hewson, and D. J. G. Crow, Magnetic field induced quantum criticality and the Luttinger sum rule, Phys. Rev. B **98**, 104419 (2018).

[13] K. Seki and S. Yunoki, Topological interpretation of the Luttinger theorem, Phys. Rev. B **96**, 085124 (2017).

[14] R. A. Horn and C. R. Johnson, *Matrix Analysis* (Cambridge University Press, Cambridge, 2012).

[15] K. Yang, H. Chen, Th. Pope, Y. Hu, L. Liu, D. Wang, L. Tao, W. Xiao, X. Fei, Y-Y. Zhang, H-G Luo, S. Du, T. Xiang, W. A. Hofer, and H-J. Gao, Tunable giant magnetoresistance in a single-molecule junction, Nature Commun. **10**, 1038 (2019).

[16] By the method of equation of motion we obtain the relation

$$G^{kk'}_{\tau\sigma} - G^{kd}_{\tau\sigma}\frac{1}{G^{d}_{\tau\sigma}}G^{dk'}_{\tau\sigma} = g^{k}_{\tau}\delta_{kk'}.$$

Therefore, the diagonal matrix with elements $g^k_\tau\delta_{kk'}$ is equal to $\mathbb{G}_{\tau\sigma}/G^d_{\tau\sigma}$, that is, the Schur complement of $G^d_{\tau\sigma}$[14]. Supplementary Eq. (S21) follows straightforwardly from the Schur decomposition property $\det \mathbb{G}_{\tau\sigma} = \det\left(\mathbb{G}_{\tau\sigma}/G^d_{\tau\sigma}\right) \times G^d_{\tau\sigma}$.
